# Supplementary material for: Tet2 and Tet3 cooperate with B-lineage transcription factors to regulate DNA modification and chromatin accessibility
Source: eLife. 2016 Nov 21;5:e18290. doi: 10.7554/eLife.18290 (PMC5142813; doi:10.7554/eLife.18290)
Supplement: Supplementary file 1 — DOI: http://dx.doi.org/10.7554/eLife.18290.019 [file elife-18290-supp1.docx]

**Supplementary file 1. Primer sequences.**

| Real-time PCR primers | Sequences (5' to 3') |
| --- | --- |
| Tcf3 E2A F | CCAACACTGGTGTCTCTCCCAAAG |
| Tcf3 E2A R | TCCTTTGACCCTAGCCGGACATAC |
| PAX5 F | GGGCTCCTCATACTCCATCA |
| PAX5 R | CTGCTGCTGTGTGAACAGGT |
| Pu.1 F | CTCCAAGCCATCAGCTTCTC |
| Pu.1 R | CTCCAAGCCATCAGCTTCTC |
| Ebf1 F | CATGTCCTGGCAGTCTCTGA |
| Ebf1 R | CAACTCACTCCAGACCAGCA |
| IRF4 F | CAATGTCCTGTGACGTTTGG |
| IRF4 R | GGCTTCAGCAGACCTTATGC |
| IRF8 F | CGCTCCAAACTCATTCTGGT |
| IRF8 R | ACATGCGGAAAGCCTGGT |
| IgCk F | GAT GTC TTG TGA GTG GCC CTC |
| IgCk R | CCA AAG ACA TCA ATG TCA AGT GGAA |
| Igk germ-line F | AGG AGG GTT TTT GTA CAG CCA |
| Igk germ-line R | TGG ATG GTG GGA AGA TGG AT |
| Actin F | AGA GGG AAA TCG TGCGTG AC |
| Actin R | CAA TAG TGA TGA CCT GGC CGT |
| Tet1 F | GAG CCT GTT CCT CGA TGT GG |
| Tet1 R | CAA ACC CAC CTG AGG CTG TT |
| Tet2 F | AACCTGGCTACTGTCATTGCTCCA |
| Tet2 R | ATGTTCTGCTGGTCTCTGTGGGAA |
| Tet3 F | GTCAGTGCCCCACGCTTCA |
| Tet3 R | ATGTTCTGCTGGTCTCTGTGGGAA |
|  |  |
| Ig rearrangement | **Sequences (5' to 3')** |
| Vdk (degerate primer for Vk) | GGCTGCAGSTTCAGTGGCAGTGGRTCWGGRAC |
| MAR35 | AAC ACT GGA TAA AGC AGT TTA |
| Jh4 | TCC CTC AAA TGA GCC TCC AAA |
| Vh588 (degenerate primer) | CGAGCTCTCCARCACAGCCTWCATGCARCTCARC |
| OL-110 (probe for Vh) | /5BiosG/CGTTTTATTTCCAGCTTGGTCCCC |
| Actin F | ATG GAT GAC GAT ATC GCT GC |
| Actin R | AGG AGT CCT TCT GAC CCA TTC |
| Jk1 probe | 56-FAM-TGGCTGTACAAAAACCCTCCTCACTGAAGA |
| BW-1 | GCGGTGACCCGGGAGATCTGAATTC |
| Jk1 F | GCAGCTACCCACTGCTCTGTT |

**Supplementary file 1. Primer sequences (continue)**

| ChIP qPCR primers | Sequences (5' to 3') |
| --- | --- |
| 3'Ek F | GGTAGGGAGCAGGTGTATGAGGCTT |
| 3'Ek R | TGATCAAGAAGACCCTTTTGAGGAAC |
| distal Ek F | GCAGTGCAGATGGACTTGG |
| distal Ek R | AGGGCAAATGTCTCCACATC |
| Mb1 promoter F | CCACGCACTAGAGAGAGACTCAA |
| Mb1 promoter R | CCGCCTCACTTCCTGTTCAGCCG |
| iEk F | GAGTTCTTTACCAAGAAAAACAATAG |
| iEk R | CTCTTGAAACTACTTTAGAGTCATTAAG |
| CD19 promoter F | CCTAATGCTATCCCCAGATGATA |
| CD19 promoter R | TAAATATTTTTCAGATGAGTGGG |
|  |  |
| Bisulfite sequencing primers | **Sequences (5' to 3')** |
| 3'Ek F | TTGGGAGTGTTTTATGGATTAAGATA |
| 3'Ek R | ACACCACCCAAACTATTAAAAAAAA |
| distal Ek F | TTGAAGGTGTAGGGTATAGGTTAGAT |
| distal Ek R | AACTACATACAAACCAAAATAACAAC |
